# Exploration of molecular features of PCOS with different androgen levels and immune-related prognostic biomarkers associated with implantation failure

**DOI:** 10.3389/fendo.2022.946504

**Published:** 2022-08-19

**Authors:** Qinyu Gao, Cong Ma, Shuyu Meng, Guanxiong Wang, Qiong Xing, Yuping Xu, Xiaojin He, Tianjuan Wang, Yunxia Cao

**Affiliations:** ^1^ Department of Obstetrics and Gynecology, First Affiliated Hospital of Anhui Medical University, Hefei, China; ^2^ National Health Commission (NHC) Key Laboratory of Study on Abnormal Gametes and Reproductive Tract (Anhui Medical University), Hefei, China; ^3^ Key Laboratory of Population Health Across Life Cycle (Anhui Medical University), Hefei, China; ^4^ Anhui Province Key Laboratory of Reproductive Health and Genetics (Anhui Medical University), Hefei, China; ^5^ Biopreservation and Artificial Organs, Anhui Provincial Engineering Research Center (Anhui Medical University), Hefei, China; ^6^ Molecular Pharmacology and Therapeutics, University of Minnesota, Twin Cities, MN, United States

**Keywords:** polycystic ovarian syndrome (PCOS), androgen, immune, biomarker, implantation failure, WGCNA

## Abstract

**Background:**

Polycystic ovary syndrome (PCOS), the most common heterogeneous reproductive disease afflicting women of childbearing age, has been recognized as a chronic inflammatory disease recently. Most PCOS patients have hyperandrogenism, indicating a poor prognosis and poor pregnancy outcomes. The molecular mechanism underlying PCOS development is still unknown. In the present study, we investigated the gene expression profiling characteristics of PCOS with hyperandrogenism (HA) or without hyperandrogenism (NHA) and identified immune-related factors that correlated with embryo implantation failure.

**Methods:**

PCOS and recurrent implantation failure (RIF) microarray datasets were obtained from the Gene Expression Omnibus (GEO) database. ClueGO software was used to perform enrichment analysis of differentially expressed genes (DEGs) in PCOS with varying androgen levels. The Weighted Co-Expression Network Analysis (WGCNA) was used to identify co-expressed modules and shared gene signatures between HA PCOS and RIF. Moreover, the upregulated DEGs of HA PCOS and RIF were intersected with shared gene signatures screening by WGCNA to excavate further key prognostic biomarkers related to implantation failure of HA PCOS. The selected biomarker was verified by qRT-PCR.

**Results:**

A total of 271 DEGs were found in HA PCOS granulosa cell samples, and 720 DEGs were found in NHA PCOS. According to CuleGO enrichment analysis, DEGs in HA PCOS are enriched in immune activation and inflammatory response. In contrast, DEGs in NHA PCOS are enriched in mesenchymal cell development and extracellular space. Using WGCNA analysis, we discovered 26 shared gene signatures between HA PCOS and RIF, which were involved in corticosteroid metabolism, bone maturation and immune regulation. DAPK2 was furtherly screened out and verified to be closely related with the development of HA PCOS, acting as an independent predictor biomarker of the embryo implantation failure. DAPK2 expression was negatively correlated to the embryo implantation rate (r=-0.474, P=0.003). The immune infiltration results suggested that upregulated DAPK2 expression was closely related with NK cell infiltration and macrophage M2, playing an essential role in the pathogenesis of implantation failure in HA PCOS.

**Conclusion:**

Our research revealed the expression profiling of PCOS with different androgen levels and identified DAPK2 as a critical prognostic biomarker for implantation failure in PCOS.

## Introduction

Polycystic ovary syndrome (PCOS) is one of the most common endocrine diseases affecting female fertility, with a morbidity rate ranging from 6 to 20% (1). PCOS typical clinical features include oligo- or anovulation, polycystic ovaries, and hyperandrogenism (2). Approximately half of women with polycystic ovaries suffer from hyperandrogenism (3). A cohort study of 2768 PCOS patients confirmed that hyperandrogenism was the primary symptom associated with increased impaired glucose tolerance, premature birth and adverse obstetric outcomes (4). Androgen excess leads to abdominal visceral adiposity and endocrine factors dysregulation, generating insulin resistance and hyperinsulinemia in PCOS. Accordingly, hyperinsulinemia could stimulate the ovary and adrenal glands to release a surge of androgen. This vicious cycle accelerates the progression of PCOS symptoms (5). Obesity and insulin resistance in PCOS caused by the androgens disrupt the balance of inflammatory and anti-inflammatory pathways (6, 7).Hyperandrogenism induces cell apoptosis, autophagy and endoplasmic reticulum stress resulting in follicle atresia and oocyte maldevelopment possibly giving rise to infertility in PCOS (8).

Infertility is commonly caused by the lack of endometrial receptivity, which could be impaired by androgen imbalances and abnormal androgen receptor expression in PCOS (9). A retrospective cohort study on 4083 women undergoing *in vitro* fertilization (IVF) or intracytoplasmic sperm injection (ICSI) showed no significant difference in pregnancy loss or perinatal complications between PCOS without hyperandrogenism and controls (10). Meanwhile, another population-based cohort study discovered that anti-androgen therapy also improves the prognosis of severe hyperandrogenism PCOS patients with low childbirth rates (11). It was found that hyperandrogenism stimulated endometrial subepithelial stroma and myometrium thickened, impairing female fertility (12). This abnormal endometrial stromal cell decidualization process damages endometrial receptivity, bringing about recurrent pregnancy loss (RPL) or recurrent implantation failure (RIF) in PCOS. Apparao et al. (13) found that serum androgen excess contributing to adverse pregnancy performance. 50% pregnancies of PCOS patients were reported to have recurrent spontaneous abortion, which was correlated with obesity, insulin resistance, hyperandrogenism and other metabolic dysregulations (14, 15). In addition to the endometrial stromal cell associated RIF (16) and recurrent miscarriage (17), expression of endometrial nature killer (NK) cells is also closely related to recurrent spontaneous abortion (18). Endometrium receptivity and endometrial microenvironment could be further impaired by testosterone through the NK cells in PCOS. Dehydroepiandrosterone (DHEA) has been proved to increase the number of NK cells, involving in the immunomodulatory (19). When NK cells are activated, NK cells cytotoxicity causes the implantation failure in IVF procedure patients (20). The evidence collectively indicates that hyperandrogenism closely correlates with immune dysfunction and poor prognosis in PCOS patients.

Granulosa cells have a strong endocrine capacity and mediate the stability of the ovarian follicular microenvironment (21), which is vital to primary follicle activation and oocyte development (22). As critical reproductive endocrine cells, granulosa cells could be influenced by androgen receptor expression and androgen levels, affecting female fertility (23). It has been discovered that DHT regulated the apoptotic genes and proteins expression through AR (24), which might be related with large follicles arrest and the suppress of granulosa cells proliferation in PCOS (23, 25). Abnormal granulosa cell function leads to luteal phase dysfunction, associated with embryo implantation failure. Wntless gene deletion in granulosa cells of mice was observed to enhance apoptotic gene expression in ovarian corpus luteum, resulting in recurrent miscarriage (26). Currently, few studies focus on the relationship between granulosa cell dysfunction and embryo implantation failure and few gene targets for predicting embryo implantation outcome in PCOS with different androgen levels. This study analyzed granulosa cell gene expression profiles in PCOS patients with or without hyperandrogenism from public databases. We also identified and validated potential prognostic biomarkers associated with adverse pregnancy outcomes and immunological derangement in PCOS, paving the way for a new approach to targeted treatment and improving the fertility of PCOS patients.

## Methods

### Data information and processing

We searched PCOS and RIF microarray datasets in the Gene Expression Omnibus (GEO, http://www.ncbi.nlm.nih.gov/geo/). The following were the inclusion criteria: 1) PCOS and RIF were diagnosed using normative and widely accepted criteria; 2) the samples were derived from granulosa cells of PCOS patients and endometrial tissue of RIF patients and corresponding healthy controls; 3) the datasets had to have clinical information of androgen levels and total testosterone ≥2.39 nmol/L was used to diagnose hyperandrogenism based on a cross-sectional study (27). We obtained five datasets for analysis and verification. PCOS granulosa cell datasets were GSE106724 (28), GSE114419 (29), GSE34526 (30) and GSE137684. GSE111974 (31) dataset contained 24 endometrial tissue samples from RIF patients and 24 samples from normal controls. These datasets information is shown in **Table 1**.

**Table 1:** Information of GSE Datasets.

Diseases	Subtype	GSE Dataset	Sample Type	Sample Size (n)		Testosterone level (nmol/L)		Platform
				Control	Case	Control	PCOS	
PCOS	Non-hyperandrogenism	GSE106724	Granulosa cell	4	4	1.40 (0.66)	2.06 (0.39)	GPL21096
	Non-hyperandrogenism	GSE137684	Granulosa cell	4	4	NHA	NHA	GPL17077
	Hyperandrogenism	GSE106724	Granulosa cell	4	4	1.40 (0.66)	3.55 (0.33)	GPL21096
	Hyperandrogenism	GSE114419	Granulosa cell	3	3	1.03 (0.09)	2.42 (0.47)	GPL17586
	Hyperandrogenism	GSE34526	Granulosa cell	3	7	1.42 (0.57)	5.96(1.50)	GPL570
	Hyperandrogenism	GSE137684	Granulosa cell	4	4	NHA	HA	GPL17077
RIF	–	GSE111974	Endometrial tissue	24	24	–	–	GPL17077

HA, hyperandrogenism; NHA, non-hyperandrogenism.

Data shown in testosterone level as the mean (SD).

The Affymetrix raw data were processed for background correction and quantile normalization by the affy package (32). The Illumina and Agilent raw data were processed through the limma package (33). The corresponding platform annotation documents annotated the probe-set identifiers with gene symbols. The average gene expression values were taken when multiple probes pointed to the same gene symbol by Perl programming language (5.30.2). The gene that was not expressed in any PCOS or RIF samples was removed. We selected and merged intersecting genes of PCOS granulosa cell samples from GSE106724, GSE114419, GSE34526, and GSE137684 datasets to search gene profiles characterization. We used sva package (34) to correct the batch effect of these different datasets, facilitating subsequent comparison and analysis.

#### Identification of differential expressed genes and functional enrichment analysis

On PCOS and RIF datasets, we performed differential expression analysis. To validate distinct gene expression in PCOS with hyperandrogenism, 18 samples of HA PCOS and 14 controls from GSE106724, GSE114419, GSE34526, and GSE137684 were chosen. Similarly, eight samples of NHA PCOS and eight controls from GSE106724 and GSE137684 were chosen. Differentially expressed genes (DEGs) were filtered by limma package, using *p* < 0.05 and | log2FC| > 0.6 as cut-off value of PCOS, and adjusted *p* < 0.05 and | log2FC| > 0.6 as cut-off value for RIF. Ggplot2 package (35) and VennDiagram package (36) were used to draw the volcano plots and Venn diagram to find the distinctive and shared gene signature of PCOS and RIF. Using STRING database (37), the protein-protein interaction (PPI) network of DEGs was constructed, and the network was visualized by Cytoscape (3.9.0) (38). Then, we utilized CytoHubba (39) plug-in and screened out the top 10 genes ranked by degree.

Gene ontology (GO) enrichment of biological processes (BPs), molecular functions (MFs), and cellular components (CCs) were examined by the clusterProfiler package (40) for the annotation of gene functions and the pathway enrichment analysis; *p* < 0.05 was considered statistically significant. Cytoscape ClueGo plug-in was used to classify GO terms and visualize GO analysis results in interaction groups (41). GraphPad Prism 8.4.3 software created a receiver operating characteristic (ROC) curve of hub genes to determine their ability to distinguish between normal controls and PCOS or RIF.

#### Weighted co-expression network analysis

The weighted gene co-expression network analysis (WGCNA) algorithm can identify gene modules with similar expression patterns, investigate the relationship between gene modules and diseases, and identify gene biomarkers (42). We analyzed the processing raw data through WGCNA to acquire HA PCOS and RIF-associated gene modules. The appropriate soft powers β (range: 1-20) were calculated with the pickSoftThreshold function in WGCNA package, constructing a scale-free distribution network. The adjacency matrix was then transformed into the topological overlap matrix (TOM) to detect connectivity between gene modules.

Further hierarchical clustering was carried out, and gene co-expression modules were discovered. We merged the similar modules after setting the minModuleSize to 50 and the mergeCutHeight to 0.25. Finally, we calculated the module eigengene (ME), which represents the expression pattern of each module, and the correlation between ME and clinical phenotype.

#### Analysis distribution of infiltration immune cells

CIBERSORT algorithm (43) evaluated the distribution of 22 immune cells for PCOS and RIF patients versus controls based on the normalized gene expression profile. The immune cell distribution of PCOS patients with varying androgen levels and RIF patients were compared and visualized using the corplot and vioplot software packages. The reshape2, ggpubr, and ggExtra R packages visualize gene biomarkers’ relationship with immune cells. The significance level was set at *p* < 0.05.

#### Participants’ selection and clinical information

The Ethics Committee approved the study and all experimental procedures of the First affiliated Hospital of Anhui Medical University (S20200007). The study included 25 PCOS patients (13 with hyperandrogenism and 12 without hyperandrogenism) and 13 controls who had signed informed consent. All participants were undergoing IVF or ICSI between October 2020 to December 2021 from the reproductive center of the First affiliated Hospital of Anhui Medical University. The 2003 Rotterdam criteria was used to diagnose PCOS (2), which required at least two of the following: oligo-ovulation and anovulation, biochemical or clinical hyperandrogenism and polycystic ovaries. Patients with diseases that cause hyperandrogenism or ovarian dysfunction, such as Cushing syndrome, congenital adrenal hyperplasia, androgen-secreting tumors, and 21-hydroxylase deficiency; patients with the ovarian disease or after ovarian surgery; abnormal uterine development, endometriosis, or uterine malignancy; IUA, thin endometrium or multiple uterine manipulations are excluded. The control subjects underwent IVF or ICSI because of tubal or male factor infertility. The blood samples were collected on the third to the fifth day of the menstrual cycle after fasting for 12 h. Serum basal estradiol (E2), progesterone (P), luteinizing hormone (LH), follicle-stimulating hormone (FSH), and testosterone (T) were determined by radioimmunoassay.

A standard controlled ovarian stimulation protocol was used on all participants. Participants were given 5000–10000 IU human chorionic gonadotropin when at least two follicles grew to 18 mm in diameter, and transvaginal ultrasound-guided oocyte retrieval was performed 36 h later. The granulosa cell samples were centrifuged at 5,000×g for 15 min to remove cell impurities and blood; then, the granulosa cells were collected and stored at -80 °C for subsequent studies. All embryos cultured to the blastocyst stage *in vitro*. The previous study considered a high-quality embryo as Gardner blastocyst score ≥ 3BB (44). All participants were transferred with high-quality embryos, and the pregnancy outcomes were continuously followed. According to the embryo transfer outcomes, we divided the patients into three groups: without implantation failure, with implantation failure less than three times, and with implantation failure greater than or equal to three times. The failed embryo implantation following at least three fresh or frozen cycles has been defined as RIF (45). Embryo implantation rate was calculated as embryo implantation rate = the number of gestational sacs (seen at vaginal ultrasound three to five weeks after transfer)/number of transferred embryos.

#### Quantitative reverse transcription-polymerase chain reaction

Total RNA was extracted from granulosa cells with TRIzol Reagent (Invitrogen); its concentration and purity were measured by NanoDrop 2000 (Thermo Fisher Scientific), and the reverse transcription was conducted by PrimeScript™ RT Master Mix (Promega). qRT-PCR was performed on LightCycler^®^ 480 II real-time qRT-PCR system (Roche) with SYBR Green master mix (Takara). Sequences of Primers (Tsingke, Nanjing, China) used in the study were as follows: GAPDH: forward 5’-GGAGCGAGATCCCTCCAAAAT-3’; reverse 5’ –GGCTGTTGTCATACTTCTCATGG-3’. DAPK2: forward 5’-TGCAGCCAAGTTCATCAAGAAGCG-3’; reverse 5’-ACACTAGCTCAAGGATGAGCACCA-3’. Relative gene expression was calculated with the 2^–ΔCT^ method.

#### Statistical analysis

SPSS26.0 software was used to analyze the clinical data and perform the logistic regression analysis (IBM, Armonk, NY, USA). The t-test (for normally distributed variables) or Mann-Whitney test assessed differences between two groups (for non-normally distributed variables). To compare three or more groups, a one-way analysis of variance (ANOVA) followed by the Tukey’s *post-hoc* test (for normally distributed variables) or Kruskal-Wallis test followed by Bonferroni *post-hoc* test (for non-normally distributed variables) was used. *p*-value < 0.05 is regarded as statistically significant. We divided the embryo implantation outcomes to further explore the risk factors associated with the severity of implantation failure. The patients were divided into without implantation failure, with implantation failure less than three times and with implantation failure greater than or equal to three times (RIF). The variables were ordinally scaled and were investigated using univariate and multivariate ordinal logistic regression analysis to identify predictors of embryo implantation failure.

## Results

### Identification of DEGs in PCOS with different androgen levels

**Figure 1:**
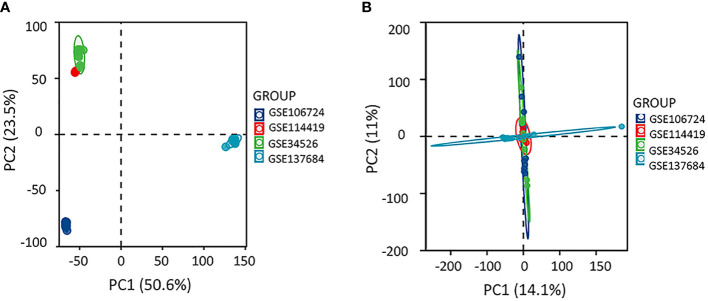
Principle-component analysis eliminating the batch effect. **(A)** The PCA plot before removing the batch effect between different datasets **(B)** The PCA plot after removing the batch effect.

**Figure 2:**
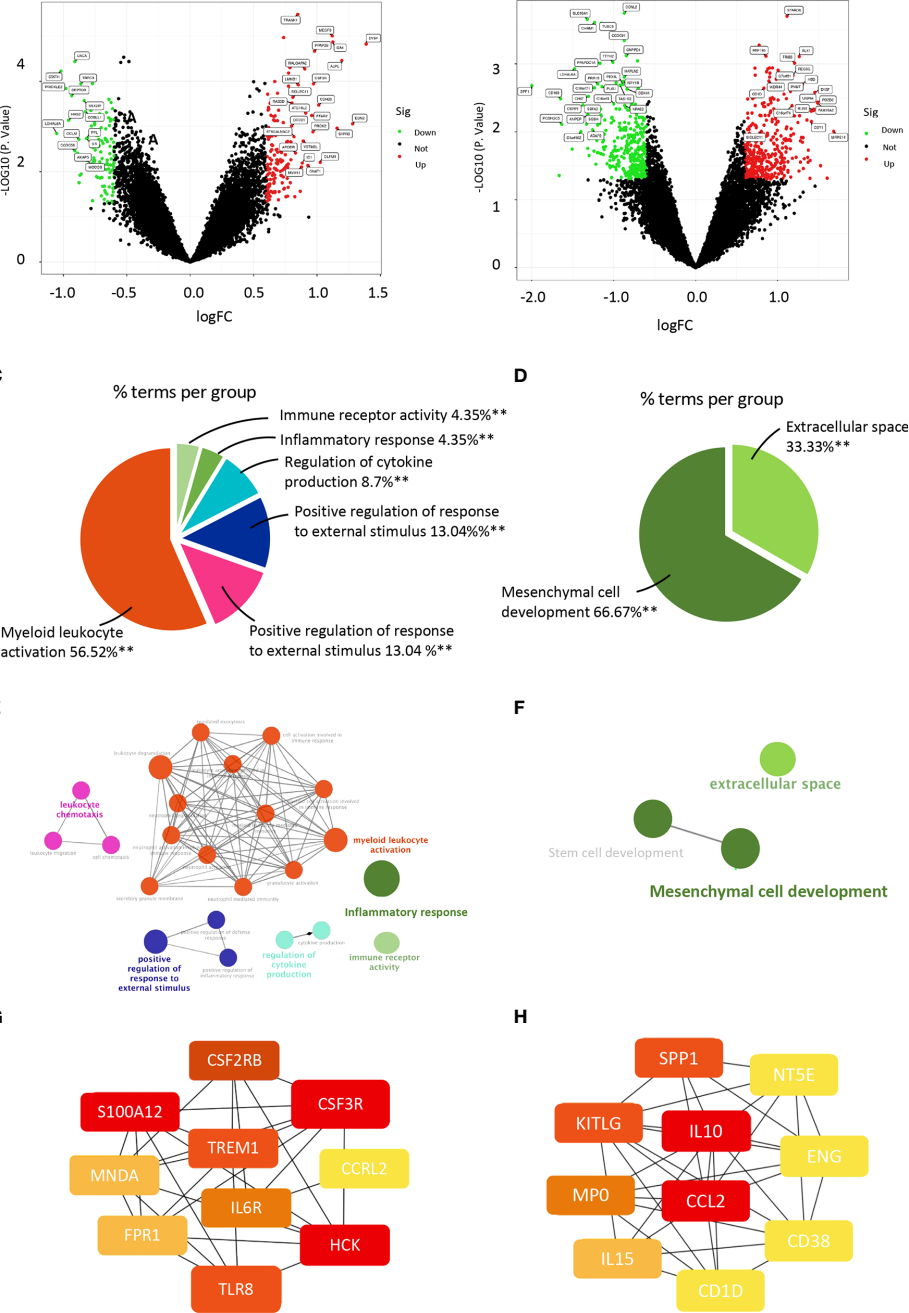
ClueGO enrichment analysis of DEGs and construction of PPI networks in PCOS with different androgen levels. **(A)** Volcano map of DEGs in the HA PCOS group and the control group. The green dots represent low expression, and the red dots represent high expression. **(B)** Volcano map of DEGs in the NHA PCOS group and the control group. The green dots represent low expression, and the red dots represent high expression. **(C)** Pie chart shows the proportion of each GO terms in HA PCOS group. **(D)** Pie chart presents the proportion of GO terms in NHA PCOS group. **(E)** The interaction network of GO terms in HA PCOS group presented by the Cytoscape plug-in ClueGO. The most significant term in each group is highlighted. **(F)** The interaction network of GO terms in NHA PCOS group presented by the Cytoscape plug-in ClueGO. The most significant term in each group is highlighted. **(G)** The top 10 hub genes ranked by degree in the PPI network of HA PCOS. **(H)** The top 10 hub genes ranked by degree in the PPI network of NHA PCOS. **P < 0.01.

We selected sequencing data from the four datasets mentioned above, merged the gene expression data, and removed the batch effect using Sva package to explore the gene profile of PCOS comprehensively. The batch effect was eliminated using principal component analysis (**Figure 1**). Samples from PCOS patients were divided into PCOS with hyperandrogenism (HA PCOS, n=18) and PCOS without hyperandrogenism (NHA PCOS, n=8) subtypes based on testosterone levels. The datasets information was summarized in **Table 1**. Then, we performed differential gene expression analysis of HA PCOS and NHA PCOS, respectively. A total of 271 DEGs were obtained for HA PCOS, including 166 upregulated and 105 downregulated genes (**Figure 2A**). A total of 720 DEGs were identified for NHA PCOS, including 384 upregulated genes and 336 downregulated genes (**Figure 2B**).

We used ClueGo to visualize the results of a GO enrichment analysis of the biological process, molecular function, and cellular component in PCOS with different androgen levels. Myeloid leukocyte activation, leukocyte chemotaxis, positive regulation of response to external stimulus, cytokine production regulation, and inflammatory response were the top five significantly enriched GO terms for HA PCOS (**Figures 2C, E**). Mesenchymal cell development and extracellular space were enriched considerably in NHA PCOS (**Figures 2D, F**). Compared with enrichment analysis of two subtypes, immune activation and inflammation were closely involved in granulosa cell dysfunction of HA PCOS. Meanwhile, DEGs in granulosa cells from NHA PCOS were closely related to cell growth and development. Go analysis results were summarized in **Table 2**. We constructed a PPI network of DEGs involved in these enrichment pathways to identify key genes. According to the degree rank, the ten core genes were TREM1, S100A12, CSF2RB, CSF3R, CCRL2, HCK, TLR8, FPR1, MNDA, and IL6R for HA PCOS. Correspondingly, IL10, CCL2, SPP1, KITLG, MP0, IL15, CD1D, CD38, ENG and NT5E was screened out for NHA PCOS. Those key genes in granulosa cells of PCOS with different androgen levels were identified and visualized (**Figures 2G, H**).

**Table 2:** GO enrichment analysis for HA PCOS and NHA PCOS.

Subtype	Ontology	ID	Term	count	P-value
HA PCOS	BP	GO:0006954	inflammatory response	34	<0.01
	BP	GO:0001816	cytokine production	25	0.02
	BP	GO:0001817	regulation of cytokine production	25	0.02
	BP	GO:0032103	positive regulation of response to external stimulus	21	0.00
	BP	GO:0031349	positive regulation of defense response	16	0.02
	BP	GO:0050729	positive regulation of inflammatory response	10	0.01
	BP	GO:0050900	leukocyte migration	19	0.04
	BP	GO:0060326	cell chemotaxis	14	0.04
	BP	GO:0030595	leukocyte chemotaxis	12	0.04
	BP	GO:0002263	cell activation involved in immune response	25	0.02
	BP	GO:0002274	myeloid leukocyte activation	26	<0.01
	BP	GO:0002366	leukocyte activation involved in immune response	24	0.05
	BP	GO:0002444	myeloid leukocyte mediated immunity	21	0.03
	BP	GO:0002275	myeloid cell activation involved in immune response	22	0.01
	BP	GO:0036230	granulocyte activation	21	0.01
	BP	GO:0002446	neutrophil mediated immunity	19	0.05
	BP	GO:0042119	neutrophil activation	21	0.01
	BP	GO:0043299	leukocyte degranulation	22	0.00
	BP	GO:0045055	regulated exocytosis	26	0.03
	BP	GO:0002283	neutrophil activation involved in immune response	19	0.04
	BP	GO:0043312	neutrophil degranulation	19	0.03
	MF	GO:0140375	immune receptor activity	9	0.03
	CC	GO:0030667	secretory granule membrane	14	0.05
NHA PCOS	BP	GO:0048864	stem cell development	13	0.03
	BP	GO:0014031	mesenchymal cell development	13	0.03
	CC	GO:0005615	extracellular space	174	0.01

### Weighted gene co-expression network construction

**Figure 3:**
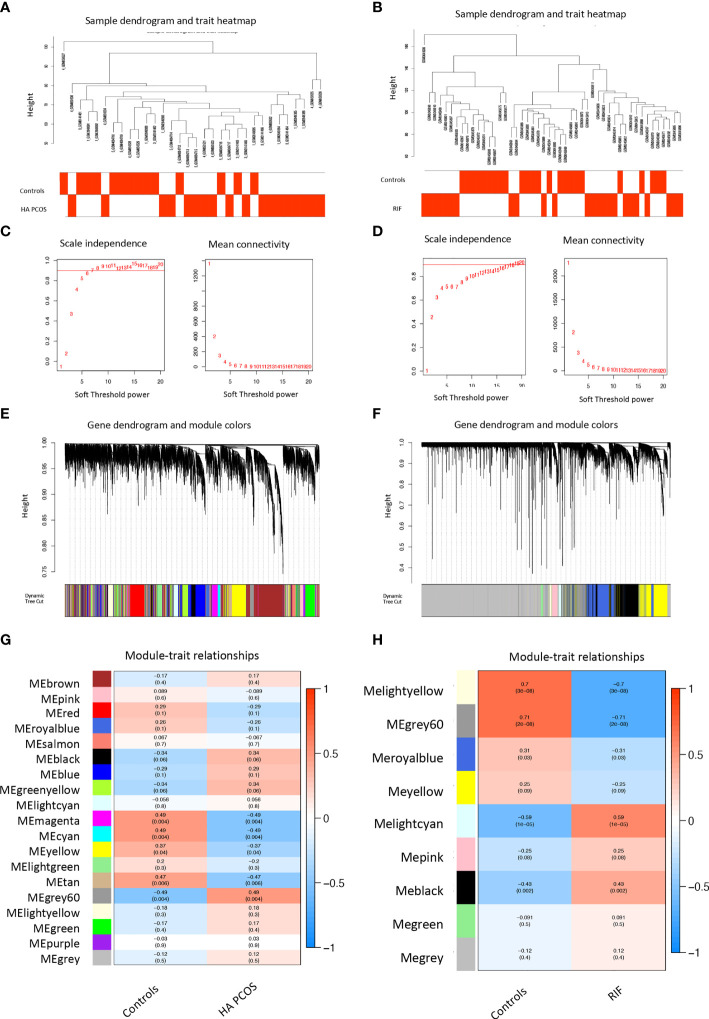
Weighted gene co-expression network analysis (WGCNA) of HA PCOS and RIF. **(A)** Sample clustering dendrogram of HA PCOS group and controls. **(B)** Sample clustering dendrogram of RIF group and controls. **(C)** Analysis of the scale-free index and mean connectivity for various threshold powers for HA PCOS. **(D)** Analysis of the scale-free index and mean connectivity for various threshold powers for RIF. **(E)** Clustering dendrogram of all DEGs in HA PCOS based on the measurement of dissimilarity (1-TOM). **(F)** Clustering dendrogram of all DEGs in RIF based on the measurement of dissimilarity (1-TOM). **(G)** Module–trait relationships in HA PCOS. The color band showed the corresponding correlation and *p*-value. **(H)** Module–trait relationships in RIF. The color band shows the corresponding correlation and *p*-value.

Androgen excess causes abnormal placental morphology and hormone metabolism, affecting trophoblast cell invasion (46). The miscarriage and obstetrical complications appearance were higher in PCOS patients with hyperandrogenism (4). To further investigate the link between HA PCOS and RIF, we used WGCNA analysis and co-expressed networks to search for shared gene expression patterns between the two diseases. The networks were built using normalized granulosa cell gene profile data (32 samples for HA PCOS analysis) and GSE111974 (48 samples for RIF analysis). We create the sample dendrogram using cluster gene expression analysis based on clinical features (**Figures 3A, B**). To ensure the network’s scale-free and competent connectivity, we set the soft threshold to 6 (R^2^ = 0.99) for HA PCOS (**Figure 3C**) and 15 (R^2^ = 0.99) for RIF (**Figure 3D**). Genes with similar expression patterns were clustered (**Figures 3E, F**). Pearson’s correlation coefficient was used to calculate the association between modules and disease, and the module-trait relationship was shown in the heatmap (**Figures 3G, H**). We obtained 19 modules in the gene profile of HA PCOS. The module “grey60” (r = 0.49 *p* = 0.004) was highly positively correlated with HA PCOS and selected to analyze further. Similarly, we obtained nine modules from the RIF dataset. The module “lightcyan” (r = 0.59 *p* < 0.001) and the module “black” (r = 0.43 *p* = 0.002) were the top two modules positively associated with RIF and were selected.

### Identification of common gene signatures and enrichment analysis

**Figure 4:**
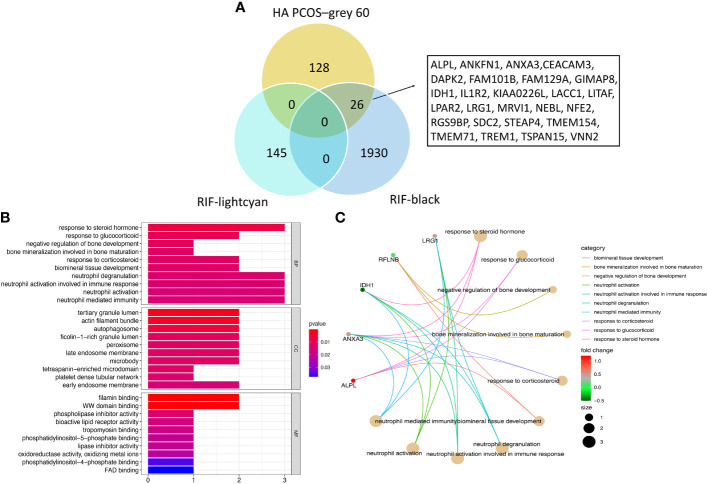
Shared gene signatures between HA PCOS and RIF. **(A)** The 26 shared gene signatures between grey60 module of HA PCOS and lightcyan and black modules of RIF. **(B)** GO enrichment analysis of biological process, molecular functions and cellular components for shared gene signatures. **(C)** Circos plot shows the relationship between genes and GO terms of biological process.

We intersected co-expressed genes from the “grey60” module of HA PCOS and the “lightcyan” and “black” modules of RIF to identify common and robust hub genes. From the “grey60” and the “black” module, 26 genes were found to be commonly shared and positively related to two diseases (**Figure 4A**). In addition, we used GO analysis to identify potential pathogenetic mechanisms associated with embryo implantation failure in HA PCOS patients. In terms of biological processes, the co-expressed 26 genes were significantly enriched in response to corticosteroids (including steroid hormone and glucocorticoid), bone mineralization, bone development regulation, immune response, and immune regulation. **Figure 4B** displays the top 10 GO terms of biological processes, cellular components, and molecular function with the most significance. The circus plot showed the expression of critical genes in relation to the enriched GO in terms of biological processes (**Figure 4C**).

### Identification of biomarkers in HA PCOS and RIF

**Figure 5:**
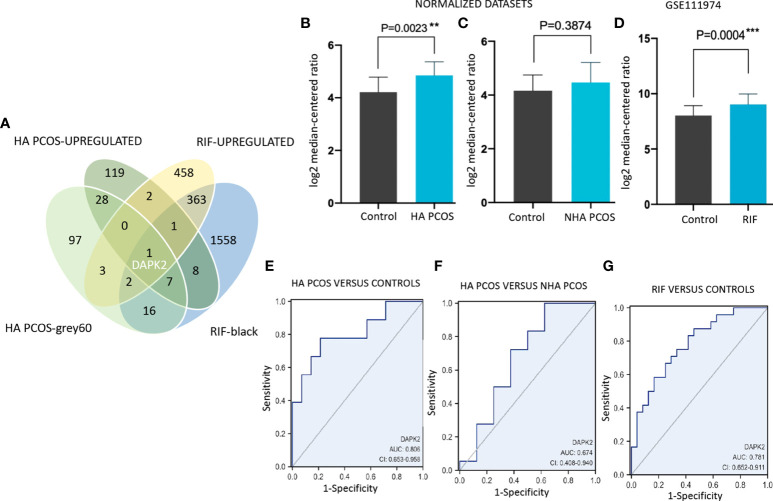
Identification and validation of DAPK2 in PCOS and RIF datasets. **(A)** Upregulated DEGs and genes of co-expressed modules in HA PCOS and RIF are intersected and DAPK2 is selected out. **(B–D)** Differential expression of DAPK2 between HA PCOS, NHA PCOS, RIF patients and controls. **(E–G)** ROC diagnostic curve for DAPK2 in HA PCOS versus controls, HA PCOS versus NHA PCOS and RIF versus controls respectively. *p < 0.01, ***p < 0.001.

For seeking key genes correlated with embryo implantation failure in HA PCOS, we identified differentially expressed genes in RIF patients (data from GSE111974). A total of 830 upregulated DEGs in RIF, 166 upregulated DEGs in HA PCOS along with 154 “grey60” module genes of HA PCOS, and 1956 “black” module genes of RIF were intersected to explore hub genes correlated with HA PCOS and RIF. DAPK2 was screened out and selected for subsequent analysis (**Figure 5A**). In the datasets mentioned above, DAPK2 expression was significantly upregulated in HA PCOS (**Figure 5B**) and there was no significant difference between NHA PCOS and controls (**Figure 5C**). DAPK2 expression also showed significant up-regulation in RIF patients compared with controls (**Figure 5D**). In addition, DAPK2 exhibited good discriminatory capability in the diagnosis of HA PCOS versus controls (AUC = 0.806, 95% CI: 0.653-0.958) and versus NHA PCOS (AUC = 0.674, 95% CI: 0.408-0.940). DAPK2 also displayed powerful diagnostic capabilities of RIF (AUC = 0.781, 95% CI: 0.652-0.911) (**Figures 5E–G**).

### Clinical Validation of DAPK2

**Figure 6:**
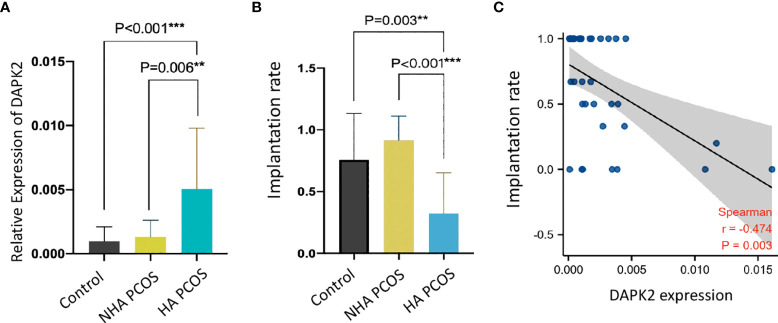
Validation of DAPK2 expression and embryo implantation rate with clinical data. **(A)** DAPK2 mRNA expression is significantly upregulated in HA PCOS compared with NHA PCOS (*p*=0.006) and controls (*p*<0.001). **(B)** the embryo implantation rate is significantly lower in HA PCOS compared with NHA PCOS (*p*=0.003) and controls (*p*<0.001). **(C)** DAPK2 expression was negatively related with embryo implantation rate. **p <0.01, ***p < 0.001.

DAPK2 mRNA expression levels in granulosa cells from PCOS and controls were confirmed with qRT-PCR in clinical samples. **Table 3** displayed the basic clinical information of patients. The findings revealed that DAPK2 mRNA expression was significantly higher in HA PCOS compared to NHA PCOS and control subjects. Furthermore, there was no statistically significant difference in DAPK2 expression between NHA PCOS and control subjects (**Figure 6A**). Moreover, the embryo implantation rate in HA PCOS was significantly lower than in PCOS without hyperandrogenism and controls (**Figure 6B**). The results indicated that PCOS with hyperandrogenism were more likely to suffer from embryo implantation failure. The association between expression of DAPK2 and embryo implantation rate was estimated by Spearman correlation analysis (**Figure 6C**), detecting that embryo implantation rate was negatively correlated with DAPK2 expression (r = -0.474 *p* = 0.003).

**Table 3:** Clinical characteristics of PCOS patients and controls.

	Controls (n=13)	PCOS with NHA (n=12)	PCOS with HA(n=13)	P. value
BMI (kg/m^2^)	21.82 (2.70)	23.81 (2.24)	23.45 (3.29)	0.17
Age (years)	28.31 (2.98)	26.33 (2.61)	29.31 (3.59)	0.65
Baseline FSH (IU/L)	10.04 (6.16)	5.82(1.75)	6.89 (1.31)	0.86
Baseline LH (IU/L)	4.92 (2.69)	6.92 (4.37)	12.42 (6.86)	<0.01 ^a,b^
Testosterone (nmol/L)	1.13 (0.43)	1.46 (0.63)	2.73 (0.54)	<0.01 ^a,b^
Endometrium thickness (mm)	11.69 (1.93)	10.18 (1.41)	9.94 (1.67)	0.13 ^a^
failed implantations, n (%)				<0.01 ^a,b^
0	8 (61.54)	10 (83.33)	1 (7.69)	
1-2	5 (38.46)	2 (16.67)	10 (76.92)	
≥3	0 (0.00)	0 (0.00)	2 (15.38)	
Embryo implantation rate (%)	75.67 (37.64)	91.67 (19.46)	32.31 (32.84)	<0.01 ^a,b^

BMI, Body mass index; LH, luteinizing hormone; FSH, follicle stimulating hormone.

Data shown as the mean (SD).

P ^a^ < 0.05 compared with control.

P ^b^ < 0.05 compared with NHA PCOS.

**Figure 7:**
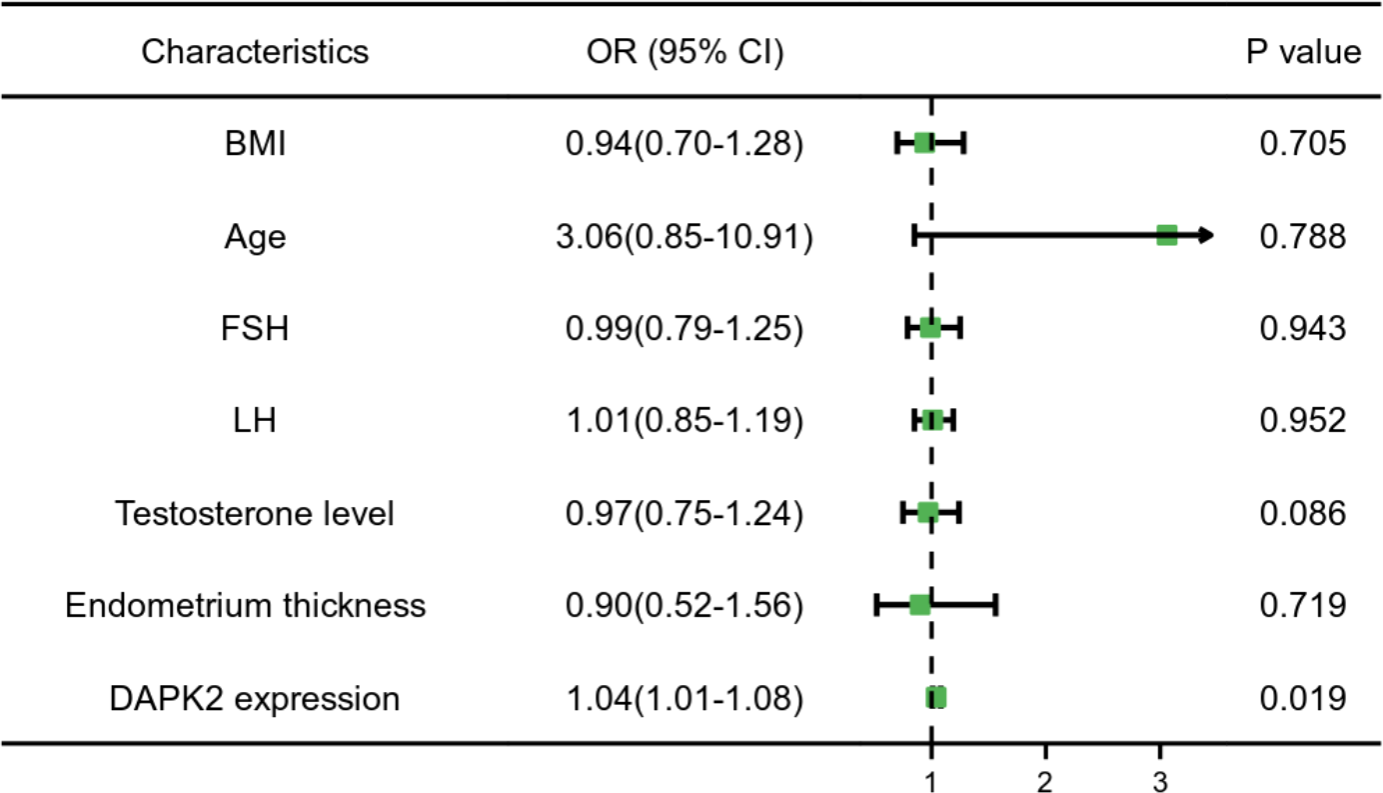
Forest plot for multivariable logistic regression analysis. *P* < 0.05 indicated that a factor is correlated with the implantation failure. OR > 1 indicated the factor was a high-risk factor. The expression of DAPK2 was observed to be an independent prognostic factor of implantation failure.

In order to further clarify the clinical significance of DAPK2, we used clinical data to identify the predictors of embryo implantation failure. BMI, age, FSH levels, LH levels, testosterone levels, endometrium thickness and DAPK2 expression were included in the univariate and multivariate ordinal logistic regression and the results was shown in **Table 4**. The multivariate logistic analysis revealed that DAPK2 (OR = 1.04, 95% CI: 1.01-1.08) was a statistically significant independent determinant of embryo implantation failure for patients undergoing assisted reproductive cycle (**Figure 7**).

**Table 4:** Univariate and multivariate logistic regression of the clinical data and DAPK2.

Variables	Univariate analysis			Multivariate analysis		
	OR	95% CI of HR	P-value	OR	95% CI of HR	P-value
BMI	1.06	0.85-1.33	0.601	0.94	0.70-1.28	0.705
Age	1.04	0.86-1.26	0.684	3.06	0.85-10.91	0.788
FSH	0.96	0.82-1.12	0.602	0.99	0.79-1.25	0.943
LH	1.13	1.01-1.28	0.042*	1.01	0.85-1.19	0.952
Testosterone level	3.90	1.56-9.78	0.004*	0.97	0.75-1.24	0.086
Endometrium thickness	0.73	0.48-1.11	0.144	0.90	0.52-1.56	0.719
DAPK2 expression	1.05	1.02-1.09	0.004*	1.04	1.01-1.08	0.019*

*p < 0.05.

### Immune cell infiltration analysis

**Figure 8:**
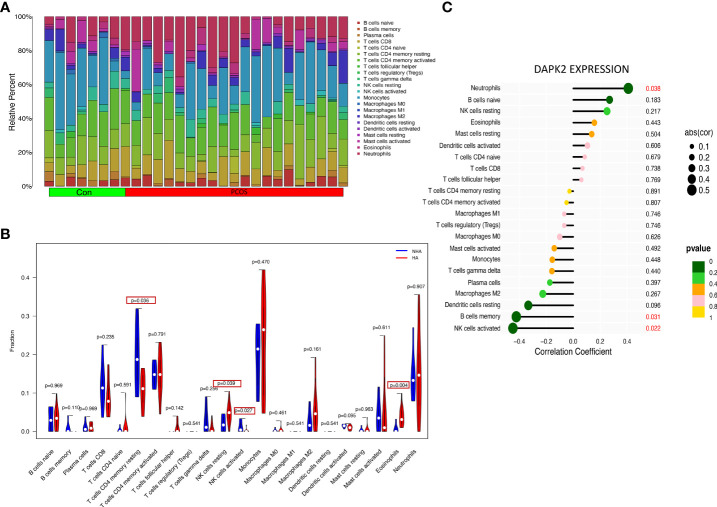
Immune cell infiltration analysis for PCOS. **(A)** The proportion of 22 immune cells in PCOS and controls. **(B)** The differential expression of 22 immune cells between HA PCOS and NHA PCOS. Blue was NHA PCOS group and red was HA PCOS group. *p* < 0.05 was framed. **(C)** The relationship between DAPK2 expression and immune cells. *p* < 0.05 was highlighted.

**Figure 9:**
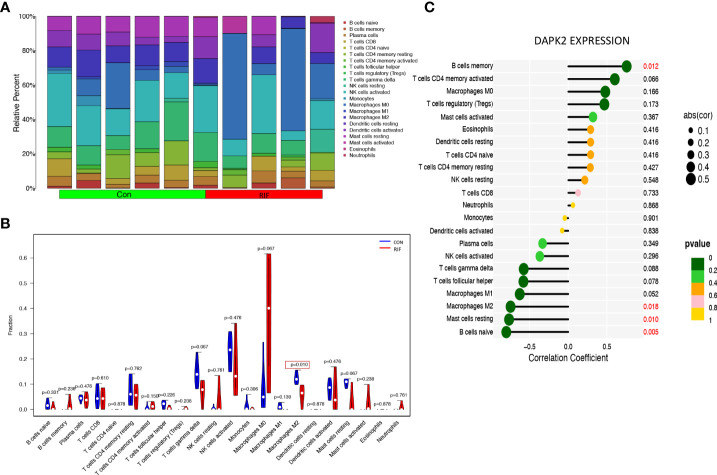
Immune cell infiltration analysis for RIF. **(A)** The proportion of 22 immune cells in RIF and controls. **(B)** The differential expression of 22 immune cells between RIF and controls. Blue was RIF group and red was control group. *p* < 0.05 was framed. **(C)** The relationship between DAPK2 expression and immune cells. *p* < 0.05 was highlighted.

The previous PCOS enrichment analysis revealed that immune-related biological processes were more enriched in HA PCOS group, which had a higher risk of developing adverse pregnancy outcomes. To identify the immune characteristics of PCOS, we used Cibersort algorithm to analyze immune cell infiltration. **Figure 8A** depicts the distribution of 22 immune cells in control subjects and PCOS patients. We detected the immune landscape difference between HA PCOS and NHA PCOS to elucidate further the relationship between immune cells and different phenotypes of PCOS. Compared with NHA PCOS, NK cell resting and eosinophils were significantly upregulated, while T cells CD4 memory resting, and NK cells activated were significantly downregulated in HA PCOS (**Figure 8B**). The correlation analysis between DAPK2 and immune cells observed that DAPK2 expression was positively correlated with neutrophils, negatively correlated with B cells memory, and NK cells activated (**Figure 8C**). Of these aspects, downregulated NK cells activated in HA PCOS are closely related to DAPK2 expression. NK cell-related immune dysfunction might play an important role in the genesis and development of PCOS. Besides, Immune cell infiltration analysis of recurrent implantation failure was shown in **Figure 9**. The distribution of immune cells was shown in **Figure 9A** and macrophages M2 was the only immune cell downregulated significantly in RIF patients (**Figure 9B**). The correlation analysis displayed that DAPK2 expression was positively correlated with B cells memory, negatively correlated with macrophages M2, mast cells resting and B cells naive (**Figure 9C**).

## Discussion

PCOS is the most common endocrine disease in women of reproductive age (1). Few studies focused on gene profiles of PCOS with different androgen levels, and the sample sizes are limited (47). High-throughput microarray or sequencing methods have provided a new perspective on exploring PCOS pathogenesis (28–31). This integrated comparative analysis described the above-identified profile, including hub genes and key pathways of granulosa cells from HA PCOS and NHA PCOS patients with different androgen levels by combining and normalizing four mRNA expression profile databases. HA PCOS differentially expressed genes are significantly enriched in immune response, consistent with previous findings that androgen excess would deteriorate immune balance in PCOS (48). Furthermore, our analysis of PPI network screened the hub genes involving in the immunity reaction and inflammation pathways of HA PCOS granulosa cells. The discovery of these specific immune-related gene signatures revealed a potential pathogenic mechanism of androgen excess in PCOS and provided a theoretical basis for PCOS anti-androgen therapy. HA PCOS patients were verified to be more susceptible to recurrent implantation failure (4, 49). However, the mechanism remains unclear. For the first time, we investigated co-expressed genes associated with the occurrence of recurrent implantation failure in hyperandrogenic PCOS. DAPK2 was demonstrated as a critical risk factor in hyperandrogenic PCOS correlated with embryo implantation failure.

The mechanism of AR in PCOS remains unclear other than AR is critical for folliculogenesis (50). Upon androgen receptor (AR) signaling, triggering receptor expressed on myeloid cells 1 (TREM1), which serves as a key gene in our PPI analysis of HA PCOS, amplifies toll-like receptor (TLR) mediated inflammation (51) and expedite cell migration (52). AR expression was highly increased in the ovaries of the DHEA-induced PCOS rat model, suggesting the potential roles of TREM1 in AR signaling and HA PCOS (53). Regarding TLR signaling, we also found the significant upregulation of TLR8 and IL6R in HA PCOS, corresponding with a previous study reporting that miR-21 enhanced TLR8 expression involved in the inflammation of PCOS (54). TLR8 and TLR7 participate in the TLR signaling pathway and recruit MyD88 to activate pro-inflammatory cytokines (IL-12, IL-6, IL-8, and TNFα) (55). There have been few studies on the role of TLR family in PCOS. We believe that TLR signaling pathway is important in the androgen-mediated inflammation process of PCOS and that it should be studied further. For NHA PCOS, some inflammatory cytokines encoded hub genes were observed to downregulate and NHA PCOS enriched pathways were more closely correlated with cell and organism development. KIT Ligand (KITLG) gene, downregulated in NHA PCOS, which has been clarified to promote primordial follicle development and ovarian folliculogenesis in ovarian granulosa cells (56). Lack of KITLG expression may lead to follicular dysplasia in NHA PCOS.

Many studies have proved that PCOS patients with hyperandrogenism have an adverse outcome in pregnancy events (4), but the mechanism remains ambiguous. Maternal androgen excess causes abnormal placental morphogenesis (46) and impaired endometrial receptivity (13), inhibiting the proliferation and promoted the apoptosis of granulosa cells (57). The occurrence of recurrent embryo implantation failure in assisted reproductive technology will increase the psychological and economic burden of PCOS patients. By WGCNA analysis, we found co-expressed gene modules based on genomic insights. 26 genes significantly positive correlated with HA PCOS and RIF were identified, which involved in corticosteroid metabolism, bone maturation and immune regulation. Androgen excess and inflammatory cytokines dysregulation affect bone metabolism and increase osteoclastic bone resorption in PCOS (58). It has been confirmed that the lower expression of follistatin induces the impair of bone morphogenetic protein signaling, impeding the process of embryo attachment to endometrium (59). Thus, hyperandrogenism may lead to the occurrence of implantation failure through the dysregulated bone metabolism in PCOS.

We then used immune infiltration analysis to uncover immune characteristics of PCOS with varying androgen levels and RIF. Abnormal distribution of NK cells causes the dysregulation of cytokines they secrete and impairs the endometrial receptivity of PCOS. NK cells, stimulated by inflammatory cytokines such as INF-γ, and TNF-α, are critical in the innate and adaptive immune response to intracellular stress, tumor, or viral infection. The previous investigation proved that testosterone and its metabolites suppressed NK cell proliferation (60) and impeded NK cell recruiting (48)which were in consistent with our study. INF-γ has been found to promote NK cell maturation, initiate maternal vasodilation and enhance blood flow to the implantation site, facilitating pregnancy (61). Consistently, memory T lymphocytes were detected to reduce in theca layer of PCOS (62), and CD4(+) memory T cells were proved to proliferate and secrete INF-γ preventing tumor growth (63). Eosinophilic granulocytes, IL-6, and TNF-α were significantly upregulated in PCOS peripheral blood, whereas lymphocytes were enriched in the ovaries of PCOS patients (64). Correspondingly, the disorder of those immune cells and cytokines in PCOS induces hyperandrogenism (65), follicle atresia, and luteal insufficiency (66). For RIF, macrophage M2 was observed with a significantly down-regulation versus controls, which might cause inadequate granulocyte colony-stimulating factor (G-CSF) release from M2 macrophage, impeding the normal process of trophoblasts invasion and migration (67).

Death-associated protein kinase 2 (DAPK2) was identified and validated as an independent decisive factor associated with implantation failure in HA PCOS, implying poor outcomes. We found that NK cell inactivation negatively related with DAPK2 expression, playing an important role in HA PCOS through the immune infiltration analysis. While down-regulation of macrophage M2 related with the overexpression of DAPK2 in RIF. As a calcium/calmodulin-dependent protein kinase, DAPK2 regulates apoptosis, cell motility, and autophagy *via* phosphorylation and mTORC1 signaling pathways (68, 69). Apoptosis of ovarian granulosa cells has been found to related with delayed oocyte maturation, affecting embryo development (70, 71), while autophagy of PCOS could cause follicular atresia and luteal insufficiency (72). Luteal function provides the necessary support for early pregnancy and luteal support defect is closely associated with implantation failure and abortion (73). Autophagy is also important for the differentiation, viral clearance and memory formation of NK cells (74). DAPK2 expression was observed to significantly elevate in granulosa cells of pig atresia follicles (75), inducing ovarian granulosa cell apoptosis (76). Researches have revealed that death-associated protein (DAP) kinase, which has 52% homology with DAPK2 (77), was involved in apoptosis induced by TNF-α and INF-γ and the down-regulation of DAP kinase resulted in resistance to cell apoptosis (78). Therefore, autophagy and apoptosis process mediated by abnormal expression of DAPK2 in PCOS might cause abnormal recruitment of NK cells, impaired folliculogenesis, implantation failure and other poor pregnancy outcomes.

The present study had some limitations that must be mentioned. First, granulosa cells have been reckoned to play an important role in PCOS pathogenesis. Due to a lack of basal androgen level information in the gene expression profiling data of blood, adipose tissue, muscle tissue, oocytes, and other tissues from PCOS patients, our analysis only focused on the sample of PCOS granulosa cells, resulting in an inadequate understanding of PCOS pathogenesis. More comprehensive studies will follow in the future to clarify the roles of androgen in the development of PCOS. Furthermore, we discovered and confirmed the expression of DAPK2 in PCOS and its relationship with embryo implantation failure. However, more *in vivo* and *in vitro* experiments are required to illuminate the specific functions of DAPK2 in immunoregulation disorder, metabolism abnormalities, corpus luteum dysfunction and miscarriage of PCOS, reinforcing the theoretical foundation of target therapy for promoting the PCOS prognosis.

Finally, this study discovered different gene profiling characteristics in PCOS granulosa cells with varying androgen levels, revealing potential mechanisms involved in the pathogenesis of different PCOS phenotypes. DAPK2 was identified and validated as a critical biomarker associated with the immune disorder and granulosa cell dysfunction in PCOS with hyperandrogenism, acting as an independent predictor of embryo implantation failure.

## Data availability statement

The original contributions presented in the study are included in the article/supplementary material. Further inquiries can be directed to the corresponding authors.

## Ethics statement

The studies involving human participants were reviewed and approved by The Ethics Committee of the First affiliated Hospital of Anhui Medical University (S20200007). The patients/participants provided their written informed consent to participate in this study.

## Author contributions

YC and TW revised and developed the outline of the manuscript. QG analyzed data, performed most of experiment and wrote the manuscript. CM and GW edited figures and performed part of experiment. SM, QX, YX and XH designed and edited the manuscript. All authors contributed to the article and approved the submitted version.

## Funding

Natural Fund of the Anhui Provincial Science and Technology Department (No. 2008085QH356) Basic and Clinical Cooperative Research and Promotion Program of Anhui Medical University (2021xkjT031) the Nonprofit Central Research Institute Fund of Chinese Academy of Medical Sciences (2019PT310002).

## Conflict of interest

The authors declare that the research was conducted in the absence of any commercial or financial relationships that could be construed as a potential conflict of interest.

## Publisher’s note

All claims expressed in this article are solely those of the authors and do not necessarily represent those of their affiliated organizations, or those of the publisher, the editors and the reviewers. Any product that may be evaluated in this article, or claim that may be made by its manufacturer, is not guaranteed or endorsed by the publisher.
